# Assessment of the relevance of the antibiotic 2-amino-3-(oxirane-2,3-dicarboxamido)-propanoyl-valine from *Pantoea agglomerans* biological control strains against bacterial plant pathogens

**DOI:** 10.1002/mbo3.43

**Published:** 2012-10-30

**Authors:** Ulrike F Sammer, Katharina Reiher, Dieter Spiteller, Annette Wensing, Beate Völksch

**Affiliations:** 1 Institute for Microbiology, Microbial Communication, University of JenaNeugasse 25, D-07743, Jena, Germany; 2 Department of Biology, Chemical Ecology/Biological Chemistry, University of KonstanzUniversitätsstr. 10, D-78457, Konstanz, Germany; 3 Julius-Kühn-Institut, Federal Research Centre of Cultivated Plants, Institute for Plant Protection in Fruit Crops and ViticultureSchwabenheimer Str. 101, D-69221, Dossenheim, Germany

**Keywords:** Environmental microbiology, microbial ecology, plant–microbe interactions, secondary metabolites

## Abstract

The epiphyte *Pantoea agglomerans* 48b/90 (Pa48b) is a promising biocontrol strain against economically important bacterial pathogens such as *Erwinia amylovora*. Strain Pa48b produces the broad-spectrum antibiotic 2-amino-3-(oxirane-2,3-dicarboxamido)-propanoyl-valine (APV) in a temperature-dependent manner. An APV-negative mutant still suppressed the *E. amylovora* population and fire blight disease symptoms in apple blossom experiments under greenhouse conditions, but was inferior to the Pa48b wild-type indicating the influence of APV in the antagonism. In plant experiments with the soybean pathogen *Pseudomonas syringae* pv. *glycinea* both, Pa48b and the APV-negative mutant, successfully suppressed the pathogen. Our results demonstrate that the *P. agglomerans* strain Pa48b is an efficient biocontrol organism against plant pathogens, and we prove its ability for fast colonization of plant surfaces over a wide temperature range.

## Introduction

*Erwinia amylovora* causes fire blight on different Rosaceae such as apple and pear, and it is one of the most economically relevant bacterial plant pathogens of these hosts. So far, the best pest control strategy is still the application of streptomycin, which is also a first-line antibiotic in veterinary medicine (Kumar et al. 2010). In Europe, the use of streptomycin is strongly restricted, in order to prevent residues of the antibiotic in fruits and honey. Furthermore, the development of streptomycin resistance in plant pathogens decreases efficacy (McManus et al. 2002). Therefore, alternative treatments are desirable. The use of antagonistic microbes as biological control organisms (BCOs) offers an alternative approach for environmentally friendly plant protection (Mayerhofer et al. 2009). BCOs possess properties that allow them to quickly establish a stable population on leaf surfaces and suppress plant pathogens in the competition for space, water, nutrients, and trace elements, for example, by production of antibiotics and nutrient-scavenging substances such as siderophores (Loper and Buyer 1991; Raaijmakers et al. 2002). Moreover, some BCOs interfere with the quorum-sensing processes that are necessary for expression of virulence genes of pathogens (Dong et al. 2004). BCOs are naturally occurring, nonpathogenic, epiphytic organisms and could pose a lower risk to human health than conventional chemical pesticides (Berg 2009). In biological control research, the fast-growing Gram-negative bacterium *Pantoea agglomerans* is of great interest, and three isolates are registered for fire blight control in the United States, Canada, and New Zealand, that is, *Pantoea vagans* (formerly: *P. agglomerans*) C9-1 as BlightBan C9-1™, *P. agglomerans* E325 as Bloomtime Biological™, and *P. agglomerans* P10c as BlossomBless™. *Pantoea agglomerans* strains are ubiquitous and a variety of isolates produce antibiotics such as pantocin A and B (Sutton and Clardy 2001; Jin et al. 2003), d-alanylgriseoluteic acid (AGA) (Giddens and Bean 2007), andrimid (Jin et al. 2006), and the E325 antibiotic (Pusey et al. 2008). Recently, 2-amino-3-(oxirane-2,3-dicarboxamido)-propanoyl-valine (APV) was identified from *P. agglomerans* and was found to inhibit the growth of a broad range of bacterial plant pathogens (e.g., *E. amylovora*, *Pseudomonas syringae* pathovars, *Agrobacterium tumefaciens*), and also the growth of the human pathogen *Candida albicans* and the yeast *Yarrowia lipolytica* under laboratory conditions (Sammer et al. 2009). APV has been detected in in vitro cultures of *P. agglomerans* 48b/90 (Pa48b). The so-called dapdiamides antibiotics are closely related to APV and were found in the strain *P. agglomerans* CU0119. A recent study describes the production of APV in the BCO *P. vagans* C9-1, which produces in addition the antibiotic pantocin A. The latter is associated with the suppression of *E. amylovora* in planta (Ishimaru 1985; Stockwell et al. 2002; Dawlaty et al. 2010; Kamber et al. 2012). However, the efficiency of APV in the interaction between pathogens and the antagonist has not been studied in apple blossom assays, which are close to the natural infection process. Immature pear slice assays revealed so far a weaker biocontrol efficacy of an APV-negative mutant than the wild-type *P. vagans* C9-1 (Kamber et al. 2012). APV is the only antibiotic compound produced by strain Pa48b, and it is produced in vitro in high concentrations (up to 0.9 mg/mL) (Sammer et al. 2009). Therefore, strain Pa48b is an ideal candidate to investigate the influence of APV on the biocontrol of bacterial pathogens. We performed comparative plant experiments between wild-type Pa48b and an APV-negative mutant coinoculated with *E. amylovora* in order to evaluate their biocontrol activity. Additionally, we used *Pseudomonas syringae* pv*. glycinea* (Psg) on soybean as a second plant model system to test the relevance of APV in biocontrol interactions. The successful biological control of Psg by Pa48b has been shown previously under both greenhouse and field conditions (Völksch et al. 1996; Völksch and May 2001). In previous studies, APV inhibits Psg under laboratory conditions, but its minimal inhibitory concentration is 10-times higher compared with that of *E. amylovora* (Sammer et al. 2009). Here, we address the growth inhibition capacity of APV on bacterial plant pathogens in plant experiments under greenhouse conditions.

Furthermore, we describe in this study the production profiles of APV of two *P. agglomerans* strains in detail and their relevance for their biocontrol efficacy against *E. amylovora* and Psg in plant experiments.

## Experimental Procedures

### Bacterial strains and media

Bacteria used in this study are listed in Table 1. *Pantoea agglomerans* and *Erwinia amylovora* strains were maintained on Standard I (St1; Roth, Karlsruhe) agar plates and cultured at 28°C. *Pseudomonas syringae* pv. *glycinea* was cultured and maintained on King's B agar plates (King et al. 1954). *Escherichia coli* S17-1λpir was maintained on St1 and grown at 37°C. Kanamycin was used in a final concentration of 25 μg/mL.

**Table 1:** Strains used in this study

Species	Strain designation(s)	Relevant characteristics or origin	Source or reference
*Erwinia amylovora*	Ea 7	Pear, Germany	K. Naumann^1^
	Ea 1/79	*Cotoneaster* sp., Germany	K. Geider^2^
*Escherichia coli*	S17-1λpir	hsdR, recA, pro RP4-2 (Tc::Mu;Km::Tn7) (λpir)	Lorenzo and Timmis (1994)
*Pantoea agglomerans*	48b/90	Soybean, Germany	B. Völksch
	39b/90	Soybean, Germany	B. Völksch
	Eh Y112-9/86	*Malus sylvestris*, USA	B. Völksch
	C9-1	*Malus sylvestris*, USA	K. Geider (V. O. Stockwell)
	Pa48b-A24, Pa48b-C1, Pa48b-C4, Pa48b-C6, Pa48b-1180-1, Pa48b-1180-2, Pa48b-1180-3, Pa48b-1180-7	APV-negative mutant of 48b/90, Km^R^	This study
*Pseudomonas syringae* pv. *glycinea*	8/83	Soybean, Germany	B. Völksch

1 Strain collection of the former Bundesanstalt für Züchtungsforschung an Kulturpflanzen, Aschersleben, Germany.

2 Julius-Kühn-Institut, Federal Research Centre of Cultivated Plants, Institute for Plant Protection in Fruit Crops and Viticulture, Dossenheim, Germany.

In order to quantify APV production, strains were cultivated in liquid 5b-medium (per liter demineralized water: solution A: 2.6 g KH_2_PO_4_, 5.5 g Na_2_HPO_4_, 2.5 g NH_4_Cl, 1.0 g Na_2_SO_4_; solution B: 0.1 g MgCl_2_·6 H_2_O, 0.01 g FeSO_4_·7H_2_O, 0.01 g MnSO_4_·4H_2_O, 8.8 g glucose; solution A and B were autoclaved separately and then mixed together) at 10, 18, or 28°C for 48 h.

### Agar diffusion assays (bioassays)

Indicator strain *E. amylovora* Ea7 was cultivated in liquid St1 medium overnight at 28°C. Bacterial suspension was adjusted to OD_578nm_ = 1 with sterile water, and 2 mL of this adjusted suspension was added to 48 mL 5b agar medium preincubated at 48°C and poured into sterile plates (120 mm). Pa48b mutants were spotted directly in this bioassay, incubated at 28°C for 24–48 h, and then analyzed. APV concentration supernatants derived from liquid cultures of *P. agglomerans* grown at 10, 18, and 28°C were filter sterilized, and then were quantified by applying 50 μL into holes (Ø 0.9 cm) in freshly prepared agar diffusion plates with *E. amylovora* Ea7 as the indicator strain. Inhibition zones were read after a 24-h incubation at 28°C. APV amounts were determined from a standard curve which was generated using purified APV in a range from 0.0064 to 1.6 mg/mL. The resulting standard curve is defined by the equation *y* = 0.6383ln(*x*) + 3.7575. Experiments were repeated three times with comparable results.

### DNA manipulation

APV-negative mutants were constructed by transposon mutagenesis with the mini-Tn5 donor plasmid pRL27 (Larsen et al. 2002). Kanamycin-resistant mutants were screened on agar diffusion assays for their growth inhibition activity. Transposon insertions were analyzed as described previously (Larsen et al. 2002). Transposon insertion sites were subcloned by plasposon rescue cloning and the flanking regions were sequenced by GATC-Biotech (Konstanz, Germany). Self-formed adaptor PCR (SEFA-PCR) was used to close gaps in the sequence of the biosynthesis cluster, and to analyze sequences upstream and downstream of the gene cluster (Wang et al. 2009). For alignments, Vector NTI 9.0 AlignX (Invitrogen, Life Technologies GmbH, Darmstadt, Germany) was used.

Southern blots were performed as described previously (Sambook et al. 1989) using a digoxigenin (DIG) DNA labeling and luminescent detection kit (Boehringer-Mannheim, Mannheim, Germany). A DIG-labeled DNA probe was prepared from a PCR amplification product carrying a 0.5-kb fragment of *apvD* gene from Pa48b. Hybridizations were carried out using a hybridization temperature of 60°C and two 10-min washes with 0.1× SSC (1× SSC consists of 0.15 mol/L NaCl plus 0.015 mol/L sodium citrate) and 0.1% sodium dodecyl sulfate at 60°C. Megaplasmids were prepared as described previously (Heringa et al. 2007).

### In planta experiments

#### Soybean plant experiments

Young trifoliate leaves of 14- to 18-day-old greenhouse-grown soybean plants (cv. Maple Arrow) were used for the in planta assays. Plants were kept in a growth chamber with a 14-h or 10-h photo period (26 or 20°C) and a 10-h or 14-h dark period (20 or 12°C), respectively. Cell suspensions of *P. agglomerans* (Pa39b; Pa48b) or of the mutant (Pa48b-C1) and Psg 8/83 (10^6^ cfu/mL in distilled water supplemented with 0.001% Tween80) were prepared. Each cell suspension of the strains was mixed with the cell suspension of Psg at a ratio of 5:1. As control, the suspensions of *P. agglomerans* and Psg were separately mixed with sterile water in the same ratio. The leaflets were inoculated by a pin–prick technique (May et al. 1997). After inoculation, the development of the bacterial populations was monitored after 16 h, 5, 10, 15, and 20 days. Every treatment was performed on different plants with three plants per treatment. At each sampling day, three leaflets from the three different plants were sampled. Ten spots from each leaflet were cut out. The 30 spots were bulked and bacteria were isolated as previously described (May et al. 1997). Experiments were repeated three times. In planta assays with purified APV were conducted as described previously (Braun et al. 2010). APV was purified as described previously (Sammer et al. 2009). Three days after pin–prick inoculation of soybean leaves with Psg, 5 μL of APV solution (0.7, 7.0, and 20.0 μg diluted in water, and water as control per wound) was dropped onto the infected wounds. This procedure was repeated on days 6 and 10 after Psg inoculation. For each concentration, 15 wounds on three different leaves and plants were investigated. Bacterial populations were determined before the first treatment with APV, and always 1 day after treatment. An additional sample was taken 20 days after Psg inoculation to assess the effect after an extended time period.

#### Apple blossom experiments

Newly opened apple blossoms were collected from greenhouse trees (cultivar “Alkmene”). Each detached blossom was placed in a well of a plastic tube rack filled with sterile tap water. After inoculation of the apple blossoms, these racks were placed in humid chambers and these were put into a climate chamber with an alternating 14-h light period (24°C) and a 10-h dark period (16°C). The detached apple blossoms were inoculated by placing a 5 μL droplet of a bacterial suspension on the receptacle. The bacterial suspensions consisted of *E. amylovora* Ea7 or Ea1/79 (5 × 10^3^ cfu/blossom) and Pa48b or its APV-negative mutant Pa48b-C1 (1 × 10^4^ cfu/blossom) for coinoculation treatment. Single inoculation with *E. amylovora* Ea7 or Ea1/79 (5 × 10^3^ cfu/blossom), Pa48b or its APV-negative mutant Pa48b-C1 (1 × 10^4^ cfu/blossom), and water were conducted as controls. The development of the bacterial population was evaluated after 5 days. For each treatment, 10 flowers were separately washed with 1 mL NaCl (0.9%) and colony-forming units were determined by plating. The experiment was repeated three times.

#### Immature pear slice assays

Walnut-sized pears were obtained from pear trees of the cultivar “Williams.” Pears were surface sterilized in ethanol (70%) and sliced. Slices were washed in bacterial suspensions of *P. agglomerans* Pa48b, Pa48b-C1, or Pa39b (10^8^ cfu/mL) and placed in petri dishes. After 2 h of drying, 10 μL of a suspension of *E. amylovora* Ea7 or Ea1/79 (5–5 × 10^3^ cfu/μL) was dropped onto the slices and incubated for 6 days at 18°C. Each treatment was performed with 20 biological replicates. After incubation, the slices were evaluated for their bacterial ooze formation and necrosis in a plus/minus rating (Beer et al. 1984).

### Statistical analysis

Data management and computation were performed using Microsoft Excel software (Microsoft Corporation, Redmond, WA). The mean values and the standard deviations were calculated and statistically compared by *t*-test using SigmaPlot 9.0 (Systat Software, Inc.).

## Results

### APV production

The *P. agglomerans* strains Pa48b and 39b/90 (Pa39b) have been previously shown to produce APV (Sammer et al. 2009). In order to define their APV production profile, both strains were grown in liquid culture at 10, 18, and 28°C, respectively. The amounts of APV produced by both strains were quantified by agar diffusion assays using *E. amylovora* as an indicator strain. Sterile culture supernatants of *Pantoea* strains were added, and inhibition zones were read after 24 h of incubation. Both Pa39b and Pa48b showed their maximal APV production at 10°C. The APV concentration of Pa39b (approximately 72 μg/mL) was six times lower than that produced by Pa48b (approximately 418 μg/mL). Pa39b inhibition zones in agar diffusion assays were absent at higher incubation temperatures (18 and 28°C) which correlates to the complete loss of APV production at both temperatures. Residual antibiotic activity (approximately 38 μg/mL) was detected in Pa48b at 18°C.

In addition to the experiments described above, we supplemented the standard agar diffusion assays with the addition of the APV inhibitor *N*-acetylglucosamine (50 μL of a 100 μg/μL solution) per hole. The resulting complete disappearance of the inhibition zones caused by sterile culture supernatants of Pa48b and Pa39b grown at 10°C indicates that both strains produced only one antibiotic which is active against both *E. amylovora* and Psg.

### Characterization of APV biosynthesis cluster by transposon mutagenesis

**Figure 1:**
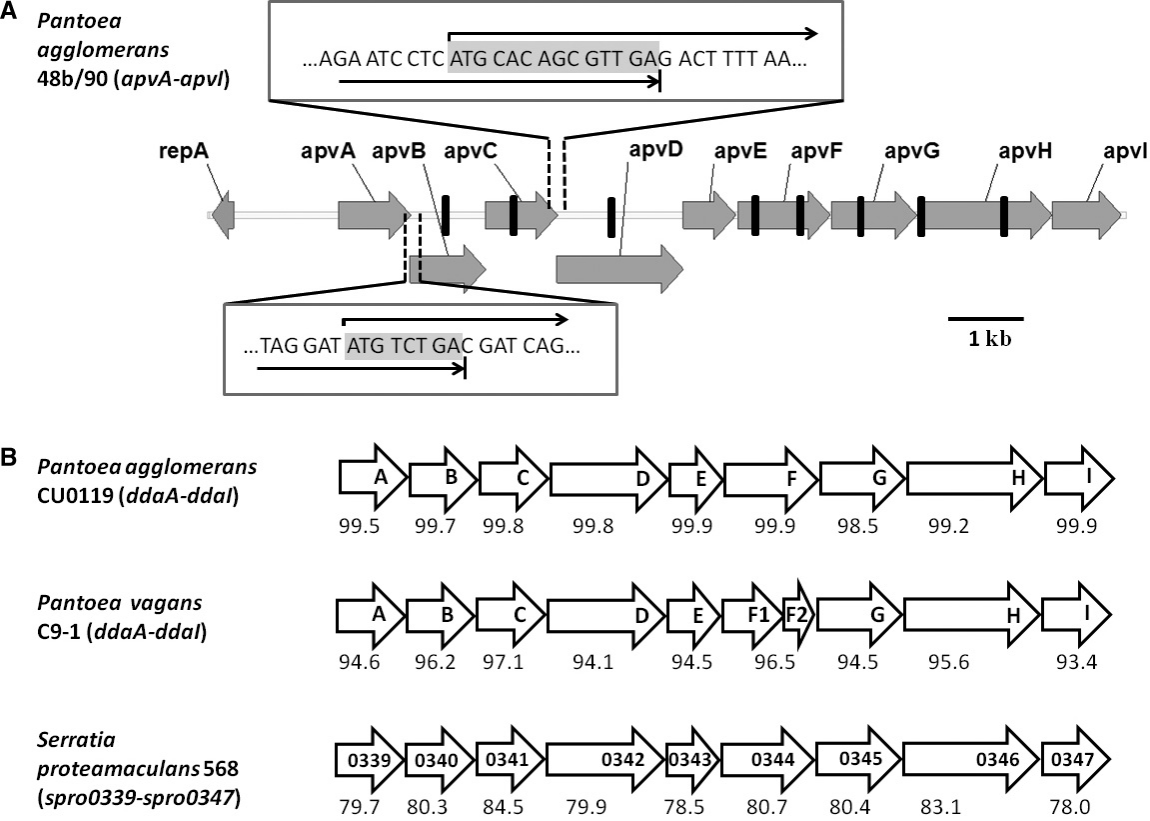
(A) APV biosynthesis cluster of Pa48b and start of *repA*, coding for a plasmid replication protein (total 12.6 kb); GenBank accession number: JQ901494; gray boxes indicate gene overlaps, black bars indicate transposon insertion sites, black arrows indicate translation start and stop of overlapping genes. (B) Comparison of APV biosynthesis cluster related gene clusters in *Pantoea agglomerans* CU0119, *Pantoea vagans* C9-1, and *Serratia proteamaculans* 568. Numbers below the clusters indicate gene identities to the APV biosynthesis cluster in Pa48b (in %).

Mini-Tn5 mutants generated according to Larsen et al. (2002) were screened for altered APV production. Eight APV-negative mutants of Pa48b were selected for their inability to inhibit *E. amylovora* in the agar diffusion assay. The respective transposon insertion sites were subcloned by plasposon rescue cloning and the flanking regions were sequenced. Insertion sites clustered in a potential biosynthetic operon. Gap-closure of the APV biosynthesis cluster in Pa48b was done by SEFA-PCR (Wang et al. 2009). Sequences were analyzed using Blastx algorithm against the NCBI database (nonredundant protein sequences) (Altschul et al. 1997). Homologies and putative function of affected genes are listed in supplementary (Table S1). Comparison with the NCBI database showed high homology to a gene cluster of unknown function in *Serratia proteamaculans* 568 and to the recently annotated herbicolin I operon in *P. vagans* C9-1, respectively (Kamber et al. 2012). In *P. agglomerans* CU0119, an annotation for dapdiamide antibiotic (DAP) biosynthesis cluster was found. The DAP biosynthesis gene cluster was heterologously expressed in *E. coli* by Dawlaty et al. (2010) and yielded five 2,3-diaminopropionate-containing antibiotics whereas Pa48b produced only APV, which is very similar to dapdiamide A and E (DAP A, DAP E) (Sammer et al. 2009; Hollenhorst et al. 2010). Alignments of the APV biosynthesis gene cluster revealed 80.7% sequence identity to the *S. proteamaculans* 568 gene cluster, 95% sequence identity to the *P. vagans* C9-1 gene cluster (Kamber et al. 2012), and 99% sequence identity to the DAP biosynthesis cluster from CU0119 (Hollenhorst et al. 2009). Further in silico analysis of potential open reading frames with Vector NTI 9.0 (Invitrogen) identified overlaps of genes *apvA* and *apvB* in 8 base pairs and in genes *apvC* and *apvD* of 14 base pairs (Fig. 1).

**Figure 2:**
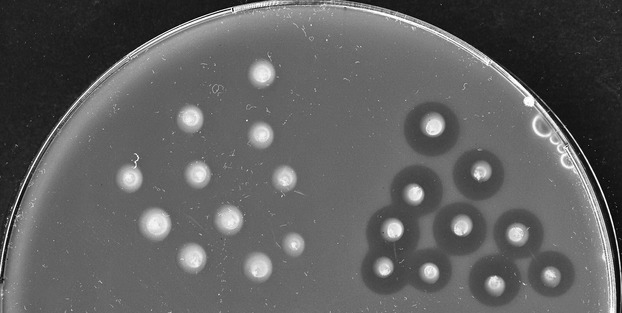
Agar diffusion assay with Ea 7 as indicator strain. Pa48b-C1 (left) was reisolated from plants after ending of the plant experiments, and the colonies were tested for their ability to produce APV. Reisolated Pa48b (right) colonies show clear inhibition zones.

One APV-negative mutant (Pa48b-C1) was used to study the function of APV. Pa48b-C1 has a transposon insertion in *apvB* coding for an ornithine cyclodeaminase, which in combination with a cysteine synthase (encoded by *apvA*) is needed for the formation of toxic l-2,3-diaminopropionate. The latter is a precursor for several antibiotics, including Zwittermicin A, DAPs, and APV (Zhao et al. 2008; Hollenhorst et al. 2010). The stability of the transposon insertion in Pa48b-C1 during plant experiments was verified by the reisolation of mutants which were tested in agar diffusion assays (Fig. 2).

### Biological control efficacy of Pa48b, Pa48b-C1, and Pa39b against bacterial plant pathogens

**Figure 3:**
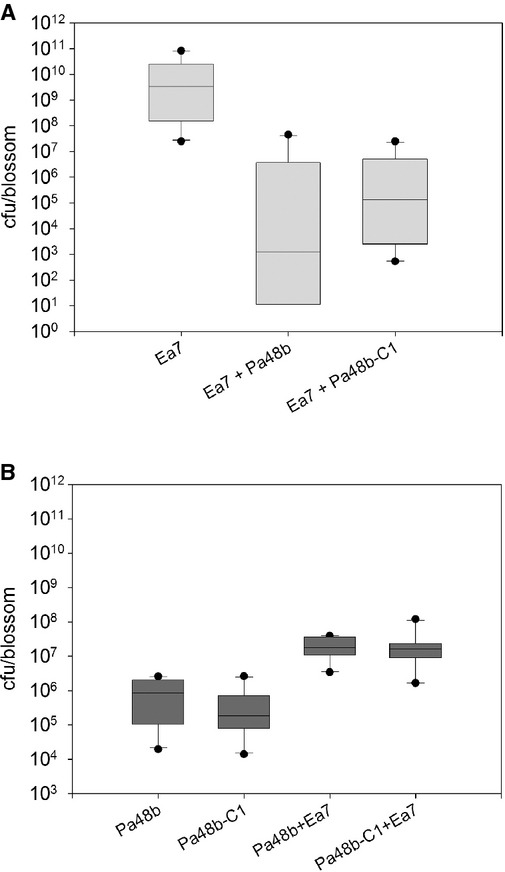
(A) Population size of *Erwinia amylovora* Ea7 after single and coinoculation with Pa48b or Pa48-C1 (after 5 days of incubation at 18°C). (B) Population size of Pa48b or Pa48-C1 after single and coinoculation with *E. amylovora* Ea7 (after 5 days of incubation at 18°C). Each box plot represents data sampled from 10 individual analyzed apple blossoms with minimal and maximal population sizes as whiskers. Interquartile (50%, five apple blossoms) in the box with the median as horizontal line.

Infection of host plants with fire blight happens by colonization of blossoms with *E. amylovora*. In laboratory experiments, fresh apple blossoms were inoculated with *E. amylovora* at 5 × 10^3^ cfu/blossom. After 5 days of incubation at 18°C infected blossoms showed typical disease symptoms. *Erwinia amylovora* was reisolated and the population density was determined to be 10^8^–10^10^ cfu/blossom. Both Pa48b and the APV-negative mutant Pa48b-C1 colonized apple blossoms and formed stable population densities of about 10^5^–10^6^ cfu/blossom in single inoculation experiments (Fig. 3B). In coinoculation experiments using *E. amylovora*, the population densities of Pa48b and Pa48b-C1 were higher (10^7^–10^8^ cfu/blossom) and the population density of *E. amylovora* was reduced by four to six orders of magnitude (Fig. 3A) compared with its single inoculation. Higher median population density values for *E. amylovora* in coinoculation with Pa48b-C1 (approximately 10^5^ cfu/blossom), compared with the coinoculation with Pa48b (approximately 10^3^ cfu/blossom), indicate an influence of APV in the antagonistic interaction. The incubation conditions in additional apple blossom experiments were adapted to a day–night cycle in order to evaluate whether these conditions lead to an increased influence of APV. Lower temperatures (about 16°C) at night were chosen to increase APV production which has been shown previously to be produced in a temperature-dependent manner in vitro (Sammer et al. 2009). Moderate temperatures (about 24°C) during the day support a fast colonization of apple blossoms. Despite the use of these adapted temperature conditions, the detected pathogen and antagonist populations were equivalent to those of constant temperature conditions (data not shown). Visual examination of the apple blossoms revealed that blossoms which were coinoculated with Pa48b or Pa48b-C1 remained symptom free by 5 days after inoculation.

Differences in the biocontrol efficiency of Pa48b, Pa48b-C1, and Pa39b against *E. amylovora* were evaluated by immature pear slice assays, which is well established for *E. amylovora* infection experiments (Bereswill and Geider 1997; Bogdanove et al. 1998; Zhao et al. 2005). Two *E. amylovora* strains which differ in their virulence (Ea7 and Ea1/79) were used in this bioassay. Immature pears were sliced and washed in suspensions of Pa48b, Pa48b-C1, and Pa39b, respectively. Afterward, an *E. amylovora* suspension was dropped onto the slices. After 6 days of incubation, symptom development was evaluated. No significant differences in the antagonistic activity of Pa48b and the APV-negative mutant Pa48b-C1 were observed (*P* < 0.05; data not shown). Thirty-five percent of the slices treated with Pa48b suspension and inoculated with the highly virulent pathogen strain Ea1/79 (5 × 10^2^ cfu/slice) developed disease symptoms, whereas only 15% of slices treated with Pa39b suspension showed necrosis. Inoculation with the low-virulent strain Ea7 (5 × 10^3^ cfu/slice) caused symptom development on 30% of the slices inoculated with Pa48b, whereas no symptoms were observed on Pa39b-inoculated pear slices. Under the tested conditions, Pa39b showed a significantly higher antagonistic activity against *E. amylovora* than either Pa48b or Pa48b-C1 (*P* < 0.05; data not shown).

**Figure 4:**
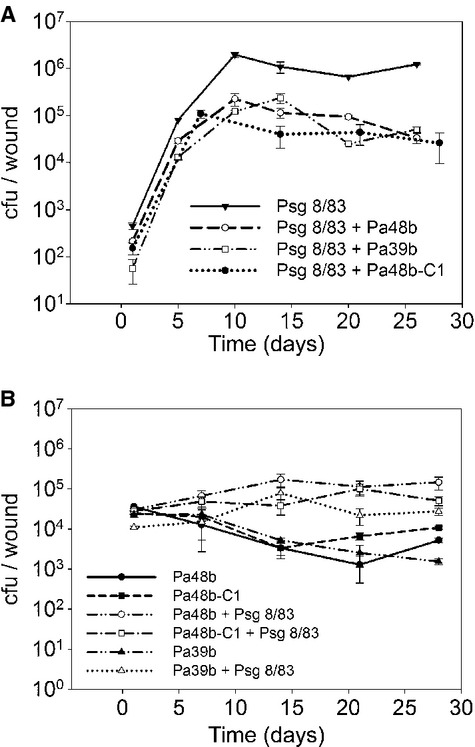
(A) Population dynamics of Psg 8/83 after single and coinoculation with Pa48b, Pa48-C1, and Pa39b on soybean leaves under moderate temperature conditions (20–26°C). (B) Population dynamics of Pa48b, Pa48b-C1, and Pa39b in single and coinoculation on soybean leaves under moderate temperature conditions (20–26°C). Single inoculations were used both to evaluate the epiphytic fitness of Pa48b, Pa48b-C1, and Pa39b, and as controls to compare with the coinoculation experiment. Each data point represents the mean value, and the bars represent the standard deviation of three replicates consisting of 10 leaf discs of one leaflet.

*Pseudomonas syringae* pv. *glycinea* (Psg) inoculation on soybean by prick-technique was used as additional plant model system in order to investigate the influence of APV. Soybean plants were cultivated under moderate (20–26°C) and low (12–20°C) temperature conditions and evaluated for disease formation in single and coinoculation experiments. The control inoculation with Psg caused typical chlorotic halos under both temperature conditions, whereas in coinoculation with Pa48b or Pa48b-C1, no chlorotic symptom formation was observed. Under moderate temperature conditions in single inoculation, the pathogen population reached about 10^7^ cfu/wound. The coinoculation of Psg with Pa48b or its APV-negative mutant Pa48b-C1 leads to a decrease of the pathogen population by two orders of magnitude at 20–26°C compared with the growth of the pathogen alone (Fig. 4A). Similar growth and population numbers of Pa48b and Pa48b-C1 were observed at 12–20°C and 20–26°C (Fig. 4B). At 12–20°C, the initial pathogen density was increased by one order of magnitude to 5 × 10^4^ cfu/wound in order to adjust for fast elimination of the pathogen by Pa48b or Pa48b-C1. This was due to the nonoptimal temperature conditions for growth of Psg. The resulting ratio was therefore 1:2 instead of 5:1 (antagonist:pathogen). Under these conditions, the high initial pathogen population allowed the pathogen to establish and spread faster compared with the lower initial pathogen populations at 20–26°C. However, Pa48b and Pa48b-C1 were able to significantly suppress Psg population by one order of magnitude at 12–20°C (*P* < 0.05; data not shown). In coinoculation experiments conducted at both 12–20°C and 20–26°C, Pa48b and Pa48b-C1 reached about 10-fold higher population sizes than occurred in single inoculations (Fig. 4B). Soybean infection experiments revealed no significant difference between Pa48b and its APV-negative mutant Pa48b-C1, at either 12–20°C or 20–26°C. Furthermore, Pa48b and Pa39b showed no differences in their biocontrol efficancy against the pathogen Psg or their epiphytic fitness (Fig. 4).

**Figure 5:**
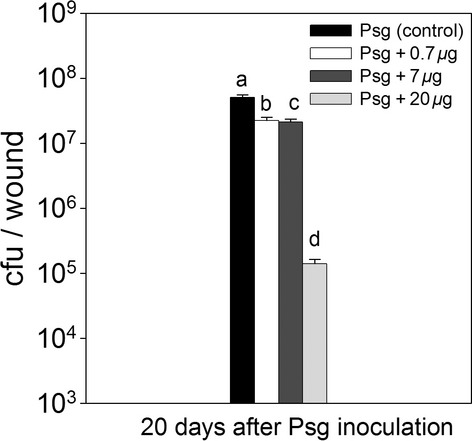
The pathogen populations after application of different amounts of purified APV (0.7, 7.0, 20.0 μg/wound, repeated three times: on day 3, 6, and 10) 20 days after Psg inoculation; values marked with the same letter did not differ significantly (*P* < 0.05; *n* = 3).

**Figure 6:**
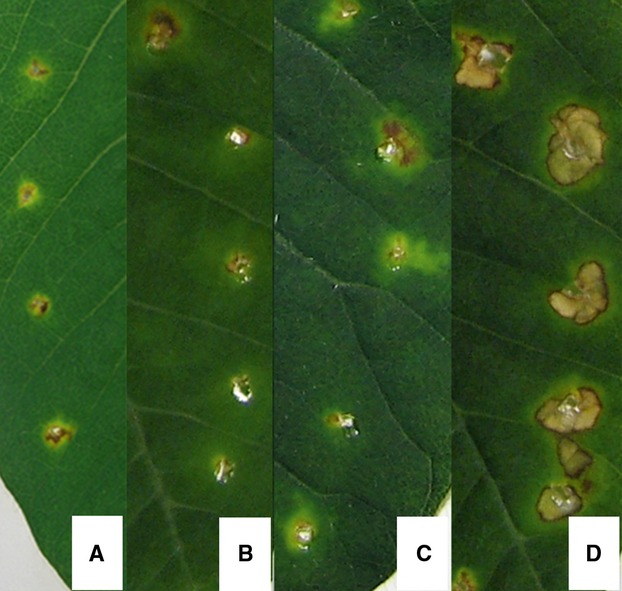
Soybean leaves 20 days after Psg inoculation. Application of water (A), 0.7 μg APV (B), 7 μg APV (C), or 20 μg APV (D) per wound. Symptoms in (D) represent a phytotoxic reaction.

In order to test for a growth inhibiting effect of APV on the pathogen population associated with soybean leaves, Psg-infected wounds were treated with purified APV. The antibiotic APV was applied at different concentrations 3 days after Psg inoculation and was added again 3 and 7 days later. The population sizes of Psg were determined on days 3, 7, 11, and 20 after inoculation. The direct application of either 0.7 or 7 μg APV per wound had only a minor effect on the growth of the pathogen, whereas application of 20 μg APV per wound resulted in a decrease of the pathogen population by about three orders of magnitude (Fig. 5). That effect was likely caused by severe damage of the leaf tissue surrounding the wounds, and was characterized by a dry parchment-like structure (Fig. 6). It is likely that the elimination of habitat and basic nutrient resources are the reasons for the decrease of the pathogen population.

## Discussion

*Pantoea agglomerans* is known to produce a variety of antibiotic molecules (Ishimaru et al. 1988; Wright et al. 2001; Jin et al. 2003, 2006). The recently described APV of Pa48b was purified and identified from several strains derived from different geographical sources (Hollenhorst et al. 2009; Sammer et al. 2009). Analysis of the APV biosynthesis gene cluster revealed high identities to the diaminopropionate-peptide biosynthesis cluster of *P. agglomerans* CU0119 (Hollenhorst et al. 2009; Dawlaty et al. 2010), *P. vagans* C9-1 (Kamber et al. 2012), and to the noncharacterized gene clusters in *S. proteamaculans* 568 (accession number: CP000826.1). The function of the genes *ddaA-I* was described as an unconventional nonribosomal peptide synthesis in the production of the DAP antibiotics of *P. agglomerans* CU0119 (Dawlaty et al. 2010). Same is postulated for *P. vagans* C9-1 (Kamber et al. 2012). DdaI is predicted to be a transmembrane efflux pump and is supposed to mediate self-resistance to the antibiotic. DdaC, an Fe(II)/α-ketoglutarate-dependent dioxygenase homolog, was shown to catalyze the epoxidation during diaminopeptide biosynthesis, which occurs only in one of the five DAPs of *P. agglomerans* CU0119 (DAP E) (Sammer et al. 2009; Hollenhorst et al. 2010) that is very similar to APV. The herbicolin I biosynthesis cluster in *P. vagans* C9-1 is located on the megaplasmid pPag2 and seems to be highly homolog to the APV biosynthesis gene cluster in Pa48b (Kamber et al. 2012). Megaplasmid preparation and Southern blot analysis using a 0.5-kb fragment of the *apvD* gene as DIG-labeled DNA probe for hybridization showed that the APV biosynthesis gene cluster is also located on a megaplasmid in Pa48b. Interestingly, antibiotics with similar chemical structures to APV occur in a range of Gram-negative (Shoji et al. 1989) and Gram-positive bacteria (Molloy et al. 1972; Cooper et al. 1988). However, the biosynthesis cluster is rarely found in the different groups, even in the genus *Pantoea* (Kamber et al. 2012). Most likely, the different strains acquired the necessary genes by horizontal gene transfer. The location of the gene cluster on a megaplasmid could have facilitated the transfer to other species (Thomas and Nielsen 2005). The sequence of the APV gene cluster was also found in the genome of *S. proteamaculans* 568, but the production of APV by this strain has not been described so far. In silico analysis of the APV biosynthesis gene cluster revealed two gene overlaps of 8 and 14 bp, respectively, which are likely to be involved in the regulation of APV biosynthesis in Pa48b. We assume that the APV biosynthesis is highly regulated on both transcriptional and translational level due to the high toxicity of the antibiotic to growing cells. The overlaps could enhance coordinated transcription and translation (Krakauer 2000) in order to ensure the coordinated biosynthesis of APV. This would prevent the accumulation of cytotoxic 2,3-diaminopropionate-containing precursor of APV (Hollenhorst et al. 2009; Dawlaty et al. 2010). Overlapping regions are not described for the DAP biosynthesis clusters of *P. vagans* C9-1 or *P. agglomerans* CU0119 neither for the gene cluster of *S. proteamaculans* 568.

To date, studies about dapdiamides have mostly addressed aspects of their biosynthesis and their mode of action (Hollenhorst et al. 2009, 2010, 2011; Dawlaty et al. 2010). Their biological functions were so far only investigated in pear slice assays by Ishimaru et al. (1988) and Kamber et al. (2012). Both showed an influence of antibiotic production on the biocontrol efficacy of *P. vagans* C9-1. Mutations that caused the loss of herbicolin I production resulted in reduced pathogen suppression on immature pear slices (Kamber et al. 2012). We assessed the possible role of APV in out-competing of other microorganisms by studying the interaction between APV nonproducing antagonists and plant pathogenic bacteria in different model systems which represent natural infection sites such as apple flowers and wounded leaves. Relevance for in planta antagonism had been shown before for other classes of antibiotics produced by *P. agglomerans*. Pantocin A and B negative double mutants of *P. agglomerans* Eh318 had a weaker biocontrol efficacy on *E. amylovora* than the wild-type. However, single mutants of Eh318 deficient in the production of either one of the two antibiotics were similarly active against *E. amylovora* as the wild type (Wright and Beer 1996). Likewise, the production of the phenazine antibiotic AGA of Eh1087 significantly enhanced the competitiveness against *E. amylovora* (Giddens et al. 2003). APV of Pa48b strongly inhibits the growth of *E. amylovora* under laboratory conditions. A single transposon insertion in *apvB* resulted in the complete loss of growth inhibition of *E. amylovora* and *Pseudomonas syringae* pv*. glycinea* in agar diffusion assays. The disruption of *apvB* leads to an absence of l-2,3-diaminopropionate, which acts as a glutamine analog in susceptible organisms. This inhibits the enzyme activity of glucosamine-6-phosphate synthase (GlmS), a key enzyme in cell wall biosynthesis. In plant experiments, apple blossoms and soybean leaves were treated with wild-type Pa48b and the APV deficient mutant Pa48b-C1 and were infected with the pathogens *E. amylovora* and *Pseudomonas syringae* pv*. glycinea,* respectively. The APV-negative mutant Pa48b-C1 still suppressed the fire blight pathogen *E. amylovora,* as well as disease symptoms on apple blossoms, but its efficacy was inferior to the wild type (Fig. 3A). The bacteriostatic effect of APV can be compensated for in vitro by supplementation with *N*-acetylglucosamine, a compound that is likely not abundant on apple blossom surfaces (Milewski 2002). Biochemical analysis of pomaceous stigma exudates showed a high quantity of sugars like glucose, fructose, and sucrose and extremely low amounts of amino acids. The predominantly found amino acids were proline, asparagines, glutamine, and serine in a total amount of 0.35 pg per flower (Pusey 2006).

In order to show the impact of purified APV on the pathogen Psg inoculated in soybean leaves, APV was applied directly onto infected wounds. The application of APV on the leaves only leads to a minor decrease of Psg population compared with nontreated Psg-infected wounds. The antibiotic may not diffuse into the leaf and therefore did not reach the pathogen inside the leaf. Additionally, Psg is less sensitive to APV than *E. amylovora* (Sammer et al. 2009). Moreover, increased concentrations of APV (20 μg/wound) caused severe damage of leaf tissue (Fig. 6). In contrast, coinoculation with Pa48b caused a significant decrease in the pathogen population in infected wounds and prevented symptom development. Furthermore, no differences in the biocontrol efficacies of Pa48b and its APV deficient mutant Pa48b-C1 on Psg on soybean leaves were observed. Under all tested temperature conditions, the antagonists equally inhibited the growth of the pathogen Psg despite their difference in APV production. Therefore, APV could not be a key factor in the antagonism. Interestingly, both Pa48b and Pa48b-C1 benefit from the coinoculation with the pathogens, *E. amylovora* or Psg, which resulted in a one to two orders of magnitude higher population compared with their single inoculation. Similar observations were described by Braun et al. (2009) for the potential biocontrol organism *Pseudomonas syringae* pv*. syringae* 22d/93 (Pss22d) in coinoculation with Psg. Pss22d grows 10-fold higher in coinoculation than in single inoculation and may cause starvation of Psg that keeps the pathogen below the threshold for disease development (Braun et al. 2009). Likewise, Pa48b and Pa48b-C1 quickly established a stable population in artificial wounds on soybean leaves and therefore represent a competitor for nutrients. The utilization of the same class of organic acids, amino acids, and carbohydrates as energy sources is likely. *Pantoea agglomerans* and *Pseudomonas syringae* have a large overlap in their spectrum of usable carbon sources (Wilson and Lindow 1994). Moreover, only 5–10% of the initial pathogen population survived the inoculation process (Braun et al. 2010). Psg, as a soybean-adapted pathogen, recovers rapidly within 1 day in single inoculations, whereas in competition with Pa48b, the pathogen population grows much slower. Interestingly, no decline of the antagonist population (Pa48b and Pa48b-C1) was observed after inoculation. Another aspect regarding nutritional competition is connected to the low iron availability on leaf surfaces and the production of iron-scavenging siderophores (Loper and Buyer 1991). Preliminary tests showed that Pa39b produces five times more of the high-affinity siderophore enterobactin than Pa48b (data not shown). Iron limitation caused by the antagonist could also be involved in pathogen suppression (Temple et al. 2004; Wensing et al. 2010) However, from this study we can conclude that the production of APV is not the key factor for *P. agglomerans* Pa48b to out-compete plant pathogens. But, APV could contribute to increase the overall fitness of *P. agglomerans*. Wensing et al. (2010) showed that the production of siderophores by antagonists support their successful and fast colonization of harsh habitats like plant surfaces. Further studies on the siderophore production of Pa48b and Pa39b will reveal the role of siderophores in the biocontrol of Psg or *E. amylovora*.

The production of secondary metabolites is a necessary tool to compete with plant pathogens secreting effector proteins (van Dijk et al. 1999) and/or phytotoxins (Nüske and Fritsche 1989; Bender et al. 1998; Barzic 1999) that are formed at 18–20°C by different *Pseudomonas syringae* pathovars typical of cold weather pathogens. Phytotoxins such as phaseolotoxin, coronatine, or persicomycin enable the pathogen to infect the plant and cause damage on plant tissue, implying a severe change in the habitat of epiphytic organisms such as *P. agglomerans*. Furthermore, temperature fluctuations between day and night could favor APV production at colder nights. Moreover, Pusey and Curry (2004) showed that *E. amylovora* colonizes apple blossoms even at temperatures between 9 and 14°C which is also the optimal temperature for APV production. Interestingly, the closely related pathogen *E. amylovora* is even more affected by Pa48b and Pa39b than Psg. In immature pear slice assays, Pa39b inhibited *E. amylovora* strains more effectively than Pa48b. Considering that Pa39b produces only minimal amounts of APV but five times more enterobactin than Pa48b, this could be involved in pathogen suppression and will be further investigated with siderophore knockout mutants. Furthermore, we showed that both *P. agglomerans* strains established stable populations on plant surfaces as quickly as the pathogens or faster. We conclude that a rapid colonization of the habitat by the antagonist in a broad temperature range is a major factor for the successful biocontrol of plant pathogens and that the production of secondary metabolites supports that process. The heterogeneity of *Pantoea* strains that was shown in this study as a result of the production of both antibiotic and siderophores could be useful for biocontrol. A new approach in the application of biocontrol organisms could be to use mixtures of different *Pantoea* strains which was also discussed by Stockwell et al. (2011). A mixture of biocontrol strains that produce different antibiotics could prevent resistance development in a pathogen population. Moreover, a broader spectrum of secondary metabolites (i.e., siderophores) that are produced by the antagonists could lead to a better adaption to environmental conditions and to reliable pathogen suppression. However, in previous field trials with mixed inocula of the BCOs *P. vagans* C9-1 and *Pseudomonas fluorescens* A506 against *E. amylovora*, it was revealed that there was an incompatibility between the two strains (Stockwell et al. 2010, 2011). An extracellular protease of *Pseudomonas fluorescens* A506 cleaves pantocin A, an antibiotic of *P. vagans* C9-1 essential for its biocontrol efficacy (Anderson et al. 2004).

We believe that a mixture of *Pantoea* strains promises less incompatibility problems. We propose for future studies to investigate these strain mixtures in order to get better suppression of fire blight and bacterial blight disease, not only in the laboratory and greenhouse but also under field conditions.

## Supporting Information

**Table S1.** Mini-Tn5 insertions in Pa48b genome.
