# Supplementary material for: Assessment of the relevance of the antibiotic 2-amino-3-(oxirane-2,3-dicarboxamido)-propanoyl-valine from Pantoea agglomerans biological control strains against bacterial plant pathogens
Source: Microbiologyopen. 2012 Oct 30;1(4):438–49. doi: 10.1002/mbo3.43 (PMC3535389; doi:10.1002/mbo3.43)
Supplement: Supplementary file 1 [file mbo30001-0438-SD1.docx]

Table S1. Mini-Tn*5* insertions in Pa48b genome.

| Mutant designation | Insertion  in Pa48b | Homologous gene  in *P. agglomerans CU0119*  (gene identity to Pa48b) | | Protein ID  (Acession number) |
| --- | --- | --- | --- | --- |
| A24 | *apvC* | *ddaC (99.5 %)* | Fe(II)/α-ketoglutaratedependent dioxygenase homologue (ADN39482.1) | |
| C1 | *apvB* | *ddaB (99.7 %)* | Rossmann-fold NAD(P)(+)-binding protein (ADN39481.1) | |
| C4 | *apvH* | *ddaH (99.2 %)* | Asparagine synthase ( ADN39485.1) | |
| C6/  1180-2 | *apvF* | *ddaF (99.9 %)* | ATP-grasp-like protein (ADN39488.1) | |
| 1180-3 | *apvD* | *ddaD (99.8 %)* | Amino acid adenylation domain containing protein (ADN39483.1) | |
| 1180-1/  1189-7 | *apvG* | *ddaG (98.5%)* | Acetyl-CoA synthetase  (ADN39487.1) | |
